# Specialty Training’s Organizational Readiness for curriculum Change (STORC): development of a questionnaire in a Delphi study

**DOI:** 10.1186/s12909-015-0408-0

**Published:** 2015-08-05

**Authors:** Lindsay Bank, Mariëlle Jippes, Scheltus van Luijk, Corry den Rooyen, Albert Scherpbier, Fedde Scheele

**Affiliations:** 1 Sint Lucas Andreas Hospital, Jan Tooropstraat 164, 1061 AE Amsterdam, Netherlands; 2 Diakonessenhuis Utrecht, Bosboomstraat 1, 3582 KE Utrecht, Netherlands; 3 Royal Dutch Medical Association (KNMG), Mercatorlaan 1200, 3528 BL Utrecht, Netherlands; 4 Maastricht UMC+, Postbus 5800, 6202 AZ Maastricht, Netherlands; 5 VU Medical Centre, Van der Boechorststraat 7, 1081 BT Amsterdam, Netherlands; 6 Athena Institute for Transdisciplinary Research, De Boelelaan 1081, 1081 HV Amsterdam, Netherlands

**Keywords:** Organizational readiness for change, Postgraduate medical education, Curriculum change, Delphi study, Questionnaire

## Abstract

**Background:**

In postgraduate medical education (PGME), programs have been restructured according to competency-based frameworks. The scale and implications of these adjustments justify a comprehensive implementation plan. Organizational Readiness for Change (ORC) is seen as a critical precursor for a successful implementation of change initiatives. Though, ORC in health care settings is mostly assessed in small scale settings and in relation to new policies and practices rather than educational change. Therefore our aim with this work was to develop an instrument to asses Specialty Training’s Organizational Readiness for curriculum Change (STORC).

**Methods:**

A Delphi procedure was conducted to examine the applicability of a preliminary questionnaire in PGME, which was based on existing instruments designed for business and health care organizations. The 41 panellists (19 trainees and 22 supervisors from 6 specialties) from four different countries who were confronted with an apparent curriculum change, or would be in the near future, were asked to rate the relevance of a 89-item web-based questionnaire with regard to changes in specialty training on a 5-point Likert scale. Furthermore, they were invited to make qualitative comments on the items.

**Results:**

In two rounds the 89-item preliminary questionnaire was reduced to 44 items. Items were either removed, kept, adapted or added based on individual item scores and qualitative comments. In the absence of a gold standard, this Delphi procedure was considered complete when the overall questionnaire rating exceeded 4.0 (scale 0–5). The overall item score reached 4.1 in the second round, meeting our criteria for completion of this Delphi procedure.

**Conclusions:**

This Delphi study describes the initial validating step in the development of an instrument to asses Specialty Training’s Organisational Readiness for curriculum Change (STORC). Since ORC is measured on various subscales and presented as such, its strength lies in analysing these subscales. The latter makes it possible for educational leaders to identify and anticipate on hurdles in the implementation process and subsequently optimize efforts for successful curriculum change.

**Electronic supplementary material:**

The online version of this article (doi:10.1186/s12909-015-0408-0) contains supplementary material, which is available to authorized users.

## Background

Around the world postgraduate medical education (PGME) programs have been restructured according to competency-based frameworks [3, 16, 23, 27, 28]. This transition is driven by altering social needs and the wish for a more patient-centred care [18, 21, 27]. Newly designed postgraduate programs embrace frameworks such as the seven roles of CanMEDS [3, 7] or the general competencies of the Accreditation Council for Graduate Medical Education (ACGME) [1]. Although the scale and implications of these reforms justify in-depth analysis of such implementation processes, relevant literature in this field is limited [6, 17, 23, 28].

Extensive research in other fields such as social sciences and business has determined that Organizational Readiness for Change (ORC) is a critical precursor for successful implementation of change initiatives [11, 13, 35, 36]. ORC is a multilevel, multifaceted construct. Multilevel, since it can be assessed at individual or supra-individual levels (e.g. department or organisation) [13, 35]. Multifaceted because it comprehends several facets such as psychological [13, 19] as well as structural aspects [13, 14]. When ORC is high, the staff involved are more dedicated to contribute to the proposed change process and more persistent in the event of setbacks. Conversely, when ORC is low, the staff involved are more likely to consider change as undesirable and may avoid or even resist participation [4, 11, 22, 30, 35, 36].

Even though the relevance is widely acknowledged in other fields, ORC in healthcare settings is rarely considered [12, 30, 36]. Research in this field primarily focussed on implementing changes in care practices, service delivery and individual doctors in small practices [6, 13, 30]. Less is known about factors influencing change implementation in larger health care organizations or in particular implementation of new postgraduate medical curricula [6, 17, 23]. For the latter, the implementation process itself has been described several times in recent years [16, 17, 23, 29]. It appeared that the extent to which implementation was successful differs between different educational teams [3, 17, 25]. In order to successfully achieve curriculum implementation, knowledge about factors causing those differences, such as ORC, is crucial. Instruments to assess ORC in health care settings do exist but predominately focus on implementation of new policies or practices [9, 30], rather than educational change. Furthermore, the instruments that do focus on educational change tend to concentrate on undergraduate curricula [15, 24]. During extensive research of the literature an instrument to asses ORC for changes in postgraduate curricula was not found. Postgraduate medical education is an unique setting in which patient care, teaching and learning are interconnected to each other and can’t be seen separately; i.e. PGME is a excellent example of learning in a workplace setting [2]. In teaching hospitals, PGME is completely integrated into clinical service. Therefore, adjustments made to the educational system influence the latter and could have consequences for e.g. working schedules, funding and (afforded) learning experiences [2]. The uniqueness of this particular setting emphasizes the need for an instrument adjusted to PGME.

Additionally, the scale and implications of the current reforms in postgraduate medical curricula further justifies the analysis of the implementation processes in order to optimize the chances for successful implementation. The assessment of ORC would enable educational leaders to identify gaps between their own expectations and those of other staff involved. Furthermore, it would help to detect problems or hurdles at an early stage and enable them to anticipate accordingly and prevent stagnation or even failure of the implementation process. Our aim is to take the first step in the development of an instrument to asses ORC in postgraduate medical education and optimize efforts to successfully implement curriculum change; Specialty Training’s Organisational Readiness for curriculum Change (STORC).

## Methods

### Conceptual model

Literature on ORC shows conceptual ambiguity about its definition and influencing factors. Definitions focus on either psychological factors [19], structural factors [14] or, in the majority of cases, a combination of both [13, 15, 35]. In this study, the following definition of ORC was adopted ‘the degree to which educational team members are motivated and capable to implement curriculum change’. In this definition ‘motivated’ mainly refers to the psychological factors, where ‘capable’ refers to the structural factors. The conceptual model of Holt [13] also reasons from a combination of both factors and subdivides readiness for change into 4 categories, namely psychological and structural factors on both an individual and organizational level. Psychological factors involve attitude, beliefs and intentions. They reflect the extent to which members of an organization are inclined to accept and implement a change. This covers factors such as belief that formal leaders are committed to change (individual level) as well as shared belief in and commitment to the change proposed (organizational level). Structural factors reflect the extent to which circumstances under which the change is occurring either enhance or inhibit the acceptance and implementation of change [13]. This concerns factors such as training, funding, and facilitating strategies on organizational level (clear goals/objectives, detailed implementation plan) as well as the presence of relevant expertise on individual level [6, 13, 15]. Since measurement of ORC in postgraduate medical training will be more focussed on the organizational level rather than the individual level, items concerning the latter were adapted to the organizational level, i.e. the educational teams consisting out of a program director, clinical staff members and trainees. Despite the fact that the conceptual model of Holt measures factors on both organizational and individual level, it was considered largely consistent with our views in relation to ORC in medical education and was therefore adopted and used to guide the development of STORC [13].

### Pre-Delphi procedure

In 2013, Jippes et al. [15], used the conceptual model of Holt [13], to guide the development of an instrument to measure ORC in undergraduate medical education. Their adaptation of ORC questionnaires designed for business and health care organizations resulted in 89 preliminary items possibly relevant for medical education in general. Through their subsequent Delphi procedure, the questionnaire was further tailored to undergraduate medical education; Medical School’s Organizational Readiness for curriculum Change (MORC) [15]. MORC was not considered appropriate to be adjusted for PGME because it focuses on the medical faculties to the neglect of students [15]. Since trainees are more actively involved in designing and implementing the curriculum [16, 17], their role would probably be underestimated. On the other hand, the conceptual model used to develop this instrument seems to be appropriate for the setting of PGME. Furthermore, MORC’s precursor includes items possibly relevant for ORC in medical education in general and was therefore suitable to be tailored to PGME. Therefore, to develop STORC, textual changes were made to adjust the preliminary items to PGME after which a Delphi procedure was conducted to define their relevance in this educational setting.

### Delphi procedure

The Delphi method is a structured research technique to reach consensus on a specific topic among a panel of experts through feedback of information and iteration [20, 26, 32]. The Delphi process is complete when consensus is reached [10, 34].

### Selection of Delphi panel

For this Delphi procedure, clinical staff and residents who were confronted with an apparent curriculum change, or would be in the near future, were asked to participate as panellists. The panellists were either recruited within our own network and approached by one of the authors or received an invitation to participate from one of the already recruited panellists (snowball sampling). For this reason, the total number of invited experts is unknown to the authors.

In order to get an appropriate and heterogeneous sample [20], the 41 panellists were either trainees or supervisors recruited from six different specialties (paediatrics, internal medicine, gynaecology, general surgery, plastic surgery and radiology) in four different countries (the Netherlands, United Kingdom, Canada, Slovenia) (Table 1). In all of these countries, curriculum change in specialty training is initiated using a competency-based framework [7, 27]. All panellists received instructions as well as a link to the web-based questionnaire by email. During each round several reminders were sent to kindly request non-responding panellists to evaluate the questionnaire. Additionally, after completion of the Delphi procedure, all participants received a book voucher as compensation for the time invested.

**Table 1:** Composition Delphi panel

	Delphi round 1 (*n* = 41)		Delphi round 2 (*n* = 34)	
	Trainees	Supervisors	Trainees	Supervisors
Netherlands	12	13	11	12
Canada	4	5	3	4
United Kingdom	1	3	1	1
Slovenia	2	1	1	1
Total	19	22	16	18
Gynaecology	5	6	4	6
Paediatrics	4	3	3	2
Internal medicine	2	2	1	2
Surgery	4	6	4	4
Plastic surgery	2	2	2	2
Radiology	2	3	2	2
Total	19	22	16	18

### Consensus and feedback

In each round, the panellists were asked to rank each statement based on their degree of agreement with questionnaire items using a 5-point Likert scale (not relevant—highly relevant) [15]. Furthermore, they were invited to make qualitative comments on each item [34].

Based on the level of agreement (i.e. relevance), items were either kept, eliminated, altered or added in order to gain consensus in the next round. In the absence of a gold standard, this Delphi procedure was considered complete when the overall questionnaire rating exceeded 4.0 (scale 0–5) [10]. Consensus on item level was achieved when > 70 % of the panellists scored that item as relevant and the average rating ≥ 4.0 (scale 0–5). When only 1 of these former criteria was met, the decision to either eliminate or alter the item was made based on the qualitative comments [15]. After each round, quantitative and qualitative results, as well as proposed alterations, were discussed within the research group. Feedback to the Delphi panel was provided in the form of an anonymous summary of the results together with the modified questionnaire and a request to evaluate the latter. When the overall rating of the questionnaire exceeded 4.0, the Delphi procedure was closed.

### Ethical considerations

This study was approved by the Ethical Review Board of the Dutch Association for Medical Education (NVMO). Informed consent was obtained from all panellists.

## Results

### Round 1

In the first round of this Delphi study (November 2013–May 2014), 41 panellists evaluated the 89-item preliminary questionnaire. Resulting qualitative comments focussed on textual shortcomings, redundancies and omissions. Having sufficient time to implement change was noted to be an important factor as well as communication about the change and knowledge on how to implement it. To the panellists’ opinion, these aspects weren’t emphasised enough, so new items covering these aspects were added. Additionally, panellists identified a need for an item about the integration of evaluations as part of the implementation plan. A new item was added in response. In contrast, items addressing external factors inhibiting change and extrinsic motivation were almost all excluded in this round; examples include ‘we are under too much pressure to do our job effectively’ and ‘we feel pressure to go along with this change’ respectively.

In the subscale ‘pressure to change’ both the item scores and qualitative comments concerning pressures from outside educational teams clearly differed between panellists from different countries. Furthermore, qualitative comments revealed a lack of clarity about the operational level of authorities such as ‘educational board’ and ‘accreditation authorities’. In response, the operational levels (hospital/regional/national) of all authorities were included in the questionnaire and presented to the panellists in the next round.

More general comments made clear that the level of abstraction asked from panellists was considered challenging. Mainly trainees said, evaluating questionnaire items based on its relevance to measure ORC in general rather than assessing the items concerning the ORC of their own educational team was difficult.

Based on the individual item scores and qualitative comments the preliminary questionnaire was reduced to 67 items; 29 items were removed, 9 items were adjusted and 7 items were added (Additional file 1). The overall questionnaire rating was 4.0.

### Round 2

In the second round of this Delphi study (June 2014–November 2014), a total of 34 experts (83 %) evaluated the 67-item questionnaire. Again, comments revealed the perceived difficulty of the level of abstraction asked from panellists. However, no more obscurities were mentioned concerning the subscale ‘pressure to change’. Qualitative comments as well as item scores on this topic clearly differed between panellists from different countries. Based on the item scores none of the authorities outside the educational teams met the criteria for consensus. As a result, institutions such as the ministry of health, accreditation authorities as well as educational boards on both regional and national level were excluded. Taking the qualitative comments into account, a new item labelled ‘external authorities’ was added in view of the international applicability of this instrument.

Comments also focussed on the involvement of trainees in the change process. The panellists thought their role should be emphasised more. As a result, one item was adjusted and the item ‘trainees are willing to innovate and/or experiment to improve training’ was added.

During this round 25 items were removed, 1 item was adjusted and 2 items were added (Additional file 2). The overall questionnaire rating was 4.1. As a result, the Delphi procedure was closed after this second round resulting in the final questionnaire consisting out of 44 items divided into 10 subscales (Table 2). According to 97 % of this Delphi panel, no relevant items were missing.

**Table 2:** Specialty Training’s Organisational Readiness for curriculum Change (STORC): final items after Delphi round 2

Specialty Training’s Organizational Readiness for curriculum Change (STORC)	Delphi mean	SD
**Pressure to change**		
Current pressures to implement this innovation in residency training comes from:		
1. Trainees in the program	4.2	0.9
2. Clinical teaching staff	4.2	0.9
3. Program directors	4.4	0.6
4. External authorities	New	New
**Appropriateness**		
This innovation in residency training is appropriate for the situation being addressed		
5. This change will improve the knowledge and skills of our trainees	4.5	0.7
6. This change is tailored to the needs for change within our residency training	4.1	0.9
7. This change will be an improvement over our current practices	4.2	1.0
**Necessity to change**		
There is a need for change		
8. There is a significant difference between the current state and the desired state of residency training	4.4	0.8
9. We need to improve our residency training curriculum	4.2	0.7
10. A change is needed to improve our residency training curriculum	4.1	0.8
**Management support and leadership**		
The educational board (hospital level):		
11. Is committed to this change	4.1	1.3
12. Provides the time and resources required to implement this change	4.3	1.3
**Staff culture**		
Clinical staff members:		
13. Feel a sense of personal responsibility to improve training	4.2	0.7
14. Cooperate to maintain and improve effectiveness of training	4.1	0.8
15. Are willing to innovate and/or experiment to improve training	4.1	0.7
16. Are receptive to changes in training methods	4.1	1.0
17. Share responsibility for the success of this project	4.1	0.9
18. Work together as a team	4.2	0.8
19. Discuss this change with trainees in both formal and informal situations	3.9	0.8
**The formal leader of this innovation in residency training (e.g. the program director):**		
20. Accepts responsibility for the success of this project	4.1	0.9
21. Has the authority to carry out the implementation of this change	4.4	0.8
22. Cooperates well with the clinical staff members	4.4	0.8
**Involvement in this innovation in residency training:**		
23. Formal educational leaders communicated well with us about the policy towards this change	4.0	0.8
24. Information provided about this change is clear	4.2	0.9
25. We are sufficiently consulted about the change	3.9	0.9
26. We are informed about the reasons for change	4.0	0.9
27. Trainees are willing to innovate and/or experiment to improve training	New	New
28. We have the skills that are needed to implement this change	4.0	1.0
**Project resources**		
The following are available to successfully implement this innovation in residency training:		
29. Financial resources	4.1	0.9
30. Training	4.3	0.8
31. Facilities	4.1	0.9
32. Staffing	4.1	0.8
33. Equipment and materials	4.0	0.8
34. Trainee awareness of this change	4.3	0.7
35. Incorporation of trainee needs	4.3	0.7
36. Evaluation protocol	4.1	0.8
**Clarity of mission and goals of this innovation in residency training**		
37. We understand how this change fits in with the desired competences of trainees	3.9	0.9
38. This curriculum change has clear goals and objectives	4.1	0.9
39. Our duties are clearly related to the goals of this change	3.9	0.9
**The implementation plan for this innovation in residency training:**		
40. Identifies specific roles and responsibilities	4.0	0.8
41. Clearly describes tasks and timelines	4.2	0.8
42. Includes appropriate training	4.0	1.0
43. Acknowledges our input and opinions	4.0	1.0
44. Includes a plan for improvement based on evaluations	4.1	0.9

Bold text: subscales of the questionnaire

## Discussion

The aim of this study was to take the first step in the development of an instrument to asses ORC in PGME. Using both a deductive as well as an inductive approach, this Delphi study assessed the content validity of STORC. In the unique context of PGME, where different systems and interests interconnect [2], the most important and applicable items and subscales to assess ORC were identified. Consisted with our conceptual model, both psychological and structural factors are represented in the 44 remaining items.

Since specialty training’s ORC is measured on various subscales (Table 2) and presented as such, STORC’s strength lies in analysing these subscales. At an early stage, this enables educational leaders to identify hurdles in the implementation process within their educational teams. Subsequently, targeted interventions aimed at facilitating successful curriculum change can be used. The effect of these interventions could be measured by repeated administration of STORC. Alternatively, STORC could be administered prior to implementation to explore whether psychological and/or structural preparedness exists to begin with. Since curriculum change is a worldwide topic, STORC was developed in an international setting in order to ensure international applicability.

### Comparison with MORC

Comparing STORC with its equivalent in undergraduate medical education [15], showed some interesting differences in relevant psychological factors and particularly in relation to relevant ‘pressure to change’. Firstly, in MORC, pressure to change could be subdivided into 3 groups; bottom-up (e.g. teaching staff), top-down (e.g. dean) and external (e.g. accreditation authorities). In contrast, in PGME pressure to change exerted by authorities from outside educational teams were all excluded. The lack of consensus on these items can partly be explained by PGME being differently organized around the world [8, 18, 27]. Secondly, almost all items concerning external factors inhibiting change and extrinsic motivation were excluded in STORC. In contrast, during the development of MORC, several items covering this topic were included and labelled as a separate dimension ‘external pressure’ [15]. Again, this emphasizes that external pressure is considered less relevant in PGME compared to undergraduate medical education. This phenomenon might not be as surprising in the light of adult learning theory, which suggests learning in PGME is driven by self-motivation and relevance to clinical practice [5]. If learning isn’t particularly driven by external factors, it might be that changing its framework, i.e. curriculum change, isn’t either. In addition, change or innovation itself is increasingly seen as an interactive learning process [31, 33]. Therefore, the principles of adult learning theory might indeed apply.

Finally, MORC included several items about believing in the capability to execute change successfully based on experiences in the past [15]. No items about this subject were included in STORC. The latter might be due to the constantly varying composition of the educational teams and as a result might make past experiences become less relevant. Additionally, curriculum change on this scale hasn’t been executed before and past experiences might therefore be considered lacking.

### Limitations

In the second Delphi round, a relatively large number of items were removed. This might be the result of adjustments made to subscales as well as reshuffling of items among subscales between the two Delphi rounds. Panellists may also have been involved in a different stage of curriculum change at subsequent rounds which might have influenced their answers. On the basis of the qualitative comments, it can be concluded that the level of abstraction asked from the panellists, was indeed considered challenging. However, the actual effect of this perceived difficulty cannot be precisely estimated.

As described above, 34 panellists (83 %) participated in the second round. Dropout of panellists is a problem commonly seen in Delphi studies [20, 32]. In this case, 7 participants failed to respond despite several reminders sent to kindly request them to evaluate the questionnaire. A high daily workload in a clinical setting where doctors have to combine clinical service, education and research might be one of the reasons for a lower response rate in round two. The time-lag between round one and two might be another explanation. Even though the assigned relevance to the questionnaire items in the first Delphi round did not differ between the 34 responders and the 7 non-responders, the effect of these non-responders on the final questionnaire is unclear [10].

### Future research

Administrating STORC and thereby obtaining statistical support by exploring its psychometric properties will be the next validating step. A few of the questions to answer would be: does STORC have a coherent internal consistency (exploratory and confirmatory factor analysis)? Is STORC a reliable instrument (reliability analysis)? How many respondents are needed to get a valid score (generalizability analysis)? Is ORC indeed related to the extent to which competency based curricula are implemented (predictive validity)?

Subsequently, measuring and relating an educational team’s ORC to the current curriculum changes might give an insight into what is needed to successfully implement these changes in specialty training and make a valuable contribution to the development of more evidence based implementation strategies.

Furthermore, administering STORC will provide information about its perceived fitness for implementation strategies in clinical practice, which could be seen as another validating step in itself. When STORC identifies areas on which educational teams score low on ORC, educational leaders could decide to make an intervention appropriate for the detected shortcoming; e.g. a low score on the subscale ‘involvement’, could result in a monthly meeting to discuss the upcoming change and exchange ideas. Subsequently, the educational leader could decide to administer STORC again after this intervention to evaluate whether progress is made.

## Conclusions

In conclusion, this article described a Delphi procedure, executed in an international setting, as the initial validating step in the development of an instrument to asses Specialty Training’s Organisational Readiness for curriculum Change (STORC). STORC could to be a useful instrument to measure ORC during curriculum change in PGME. Though gathering empirical data to take further validating steps and assess its psychometric properties are needed.

## Additional files

Additional file 1:
**Results Delphi round 1.**
Additional file 2:
**Results Delphi round 2.**

## Abbreviations

STORC: Specialty Training’s Organisational Readiness for curriculum Change
ORC: Organisational readiness for change
PGME: Postgraduate Medical Education
MORC: Medical School’s Organizational Readiness for curriculum Change
