# Supplementary material for: Specialty Training’s Organizational Readiness for curriculum Change (STORC): development of a questionnaire in a Delphi study
Source: BMC Med Educ. 2015 Aug 5;15:127. doi: 10.1186/s12909-015-0408-0 (PMC4525745; doi:10.1186/s12909-015-0408-0)
Supplement: Additional file 1: — Results Delphi round 1. [file 12909_2015_408_MOESM1_ESM.pdf]

Appendix A – Results Delphi round 1

Specialty Training's Organizational Readiness for curriculum Change (STORC)	Delphi mean	SD	Result
**Pressure to change**<br>Current pressures to implement this innovation in residency training comes from:			
1. Trainees in the program	4.0	1.1	Stay
2. Clinical teaching staff	4.1	1.0	Stay
3. Program directors	4.1	0.9	Stay
4. Hospital board	3.2	1.1	Change
5. Community organizations	2.9	1.2	Remove
6. Ministry of Health/Education	3.5	1.0	Stay
7. Accreditation authorities	4.0	1.0	Change
8. Educational board	3.9	0.8	Change
Professional (scientific) associations (national level)			New
**Appropriateness**<br>This innovation in residency training is appropriate for the situation being addressed.			
9. This change will have a favourable effect on our residency training	4.4	0.8	Remove
10. This change will improve the performance of our residency training	4.3	0.9	Change
11. This change is correct for our situation	3.7	0.9	Remove
12. This change will prove to be best for our situation	3.8	0.9	Remove
This change meets the required changes needed within our residency training			New
**Valence**<br>This innovation in residency training is beneficial. R = reversed question			
13. Our residency training will benefit from this change	4.3	0.9	Change
14. Our residency training will lose some valuable assets when we implement this change (R)	3.9	1.0	Change
15. This change will be an improvement over our current practices	4.4	0.8	Stay
**Discrepancy**<br>There is a significant difference between the current state and the desired state of residency training.			
16. We need to change the way we do some things in our residency training	4.1	0.8	Remove
17. We need to improve our residency training curriculum	4.1	0.7	Stay
18. A change is needed to improve our residency training curriculum	4.2	0.8	Stay

There is a significant difference between the current state and the desired state of residency training			New
**Management Support (e.g. hospital and educational board)**<br>Belief that formal and informal leaders are committed to the success of this innovation in residency training.			
19. Management encourages us to embrace this change	3.7	0.9	Remove
20. Our educational leaders are committed to this change	4.4	0.7	Stay
21. Management sends a clear signal our residency training is going to change	3.6	0.9	Remove
**Change-related interaction:**<br>This innovation in residency training is important enough for people to care about. R = reversed question.			
22. This change is a frequent topic of conversation	3.9	0.9	Remove
23. We rarely discuss this change among colleagues (R)	3.0	1.0	Remove
24. This change seems to have generated quite a bit of e-mail traffic	2.9	1.1	Remove
**Commitment to change:**<br>Shared dedication to change residency training. R = reversed question.			
25. We believe in the value of this change for our residency training	4.4	0.8	Stay
26. This change is a good strategy for our residency training	4.1	0.9	Stay
27. This change serves an important purpose	4.3	0.9	Stay
28. We have no choice but to go along with this change	3.4	1.3	Remove
29. We feel pressure to go along with this change	3.4	1.1	Remove
30. We have too much at stake to resist this change	3.4	1.0	Remove
31. We feel a sense of duty to work towards this change	3.6	0.9	Remove
32. We would feel guilty about opposing this change	2.7	1.0	Remove
33. We do not feel any obligations to support this change (R)	3.3	1.2	Remove
**Efficacy**<br>Shared capability to change residency training			
34. There are some tasks required when we change which we can't do well (R)	3.7	1.0	Remove
35. We have the skills that are needed to implement this change	4.2	0.8	Stay
36. When we set our mind to it, we can learn everything that will be required when this change is implemented	3.8	1.0	Remove
37. The past experiences make us confident that we will be able to perform successfully after this change is made	4.0	0.9	Stay
**Ability of management to lead this innovation in residency training (e.g. hospital**			

**and educational board)** Management: R = reversed question.			
38. Pays sufficient attention to the personal consequences that the changes could have	4.1	1.1	Stay
39: Speaks up for us during the change process	3.9	1.0	Remove
40: Coaches us very well about implementing this change	4.1	0.9	Stay
41: Has trouble in adapting their leadership styles to this change (R)	3.3	1.2	Remove
42. Rewards educational innovations and creativity to improve training	4.2	0.9	Stay
43. Solicits opinions of us regarding decisions about training	4.0	1.0	Stay
44. Seeks ways to improve education	4.3	0.6	Stay
**Staff culture** Clinical staff members:			
45. Have a sense of personal responsibility for improving training	4.3	0.7	Stay
46. Cooperate to maintain and improve effectiveness of training	4.3	0.7	Stay
47. Are willing to innovate and/or experiment to improve training	4.2	1.0	Stay
48. Are receptive to changes in training methods	4.2	1.0	Stay
49. Share responsibility for the success of this project	4.4	0.7	Stay
50. Have clearly defined roles and responsibilities with respect to residency training	4.1	0.8	Stay
51. Have release time or can accomplish innovations in residency training within their regular work load	4.4	0.7	Stay
52. Have staff support and other resources required for the project	4.2	0.8	Change
Have insufficient knowledge on change management to lead the implementation of this change (R)			New
Carry out the same opinion regarding this change towards trainees			New
Discuss this change with trainees in both formal and informal situations			New
**The formal leader of this innovation in residency training (e.g. the program director):**			
53. Accepts responsibility for the success of this project	4.4	0.8	Stay
54. Has the authority to carry out the implementation	4.6	0.7	Stay
55. Is considered an opinion leader	4.2	1.0	Stay
56. Cooperates well with the clinical staff members	4.6	0.6	Stay
**Clinical staff cohesiveness** R = reversed question.			
57. Some clinical staff do not do their fair share of work (R)	3.4	1.2	Remove

58. Clinical staff get along very well	3.8	0.9	Remove
59. There is too much friction among clinical staff (R)	3.5	1.2	Remove
60. The clinical staff work together as a team	4.0	0.9	Stay
61. Clinical staff are quick to help one another when needed	3.7	1.0	Remove
**Involvement in this innovation in residency training:**			
62. There is good communication between formal educational leaders and us about the policy towards this change	4.3	0.6	Stay
63. Information provided on the change is clear	4.5	0.7	Stay
64. We are sufficiently informed about the progress of the change	4.2	1.0	Stay
65. We are consulted about the change sufficiently	4.1	0.9	Stay
66. We are informed about the reasons for the changes	4.3	0.8	Stay
**Perceived stress levels**			
67. We often show signs of stress and strain	3.6	1.0	Remove
68. The heavy workload here reduces learning effectiveness	4.0	1.1	Change
69. Frustration is common here	3.4	1.1	Remove
70. We are under too much pressure to do our job effectively	3.7	1.1	Remove
**General resources**<br>For this innovation in residency training we have the necessary support in terms of:			
71. Financial resources	4.1	0.9	Stay
72. Training	4.5	0.8	Stay
73. Facilities	4.4	0.8	Stay
74. Staffing	4.4	0.6	Stay
**Project resources**<br>The following are available to successfully implement this innovation in residency training:			
75. Staff incentives (e.g. financial reward, promotion)	3.9	0.9	Change
76. Equipment and materials	4.2	0.7	Stay
77. Trainee awareness of this change	4.4	0.8	Stay
78. External advisory board	3.2	0.9	Remove
79. Incorporation of trainee needs	4.3	0.8	Stay
80. Evaluation protocol	4.3	0.8	Stay
**Clarity of mission and goals of this innovation in residency training.**			

R = reversed question.			
81. Some of us get confused about the main goals for this change (R)	3.4	1.1	Remove
82. We understand how this change fits as part of the desired competences of trainees	4.3	0.7	Stay
83. This curriculum change has clear goals and objectives	4.6	0.6	Stay
84. Our duties are clearly related to the goals of this change	4.2	0.7	Stay
85. Clinical staff has a clear plan for this change	4.2	0.7	Stay
**The implementation plan for this innovation in residency training:**			
86. Identifies specific roles and responsibilities	4.2	0.6	Stay
87. Clearly describes tasks and timelines	4.3	0.8	Stay
88. Includes appropriate training	4.3	0.7	Stay
89. Acknowledges clinical staff input and opinions	4.2	0.7	Stay
Includes a plan for improvement based on evaluations			New
