# Supplementary material for: Specialty Training’s Organizational Readiness for curriculum Change (STORC): development of a questionnaire in a Delphi study
Source: BMC Med Educ. 2015 Aug 5;15:127. doi: 10.1186/s12909-015-0408-0 (PMC4525745; doi:10.1186/s12909-015-0408-0)
Supplement: Additional file 2: — Results Delphi round 2. [file 12909_2015_408_MOESM2_ESM.pdf]

Appendix B – Results Delphi round 2

Specialty Training's Organizational Readiness for curriculum Change (STORC)	Delphi mean	SD	Result
**Pressure to change**<br>Current pressures to implement this innovation in residency training comes from:			
1. Trainees in the program	4.2	0.9	Stay
2. Clinical teaching staff	4.2	0.9	Stay
3. Program directors	4.4	0.6	Stay
4. Educational board (hospital level)	3.6	1.1	Remove
5. Ministry of Health/Education	3.5	1.2	Remove
6. Accreditation authorities (national level)	3.9	0.9	Remove
7. Educational board (regional level)	3.9	0.6	Remove
8. Professional (scientific) associations (national level)	3.9	0.9	Remove
External authorities			New
**Appropriateness**<br>This innovation in residency training is appropriate for the situation being addressed.			
9. This change will improve the knowledge and skills of our trainees	4.5	0.7	Stay
10. This change meets the required changes needed within our residency training	4.1	0.9	Stay
11. Our residency training will lead to more broadly trained doctors (e.g. management skills, relational skills, teamwork)	3.9	1.0	Remove
12. Our residency training will lose valuable parts when we implement this change ®	3.7	1.1	Remove
13. This change will be an improvement over our current practices	4.2	1.0	Stay
14. We believe in the value of this change for our residency training	3.9	0.9	Remove
15. This change is a good strategy for our residency training	3.7	0.9	Remove
16. This change serves an important purpose	3.9	1.1	Remove
**Necessity to change**<br>There is a need for change			
17. There is a significant difference between the current state and the desired state of residency training	4.4	0.8	Stay
18. We need to improve our residency training curriculum	4.2	0.7	Stay
19. A change is needed to improve our residency training curriculum	4.1	0.8	Stay
**Management Support and leadership**<br>The educational board (hospital level):			

20. Is committed to this change	4.1	0.9	Stay
21. Pays sufficient attention to the personal consequences that the changes could have	3.8	1.1	Remove
22. Coaches us very well in implementing this change	3.8	1.1	Remove
23. Rewards educational innovations and creativity to improve training	3.9	0.9	Remove
24. Solicits opinions of us regarding decisions about training	3.7	1.0	Remove
25. Seeks ways to improve training	3.8	0.8	Remove
26. Provides the time and resources required to implement this change	4.3	1.1	Stay
**Efficacy**<br>Shared capability to change residency training			
27. We have the skills that are needed to implement this change	4.0	1.0	Stay
28. The past experiences make us confident that we will be able to perform successfully after this change is made	3.6	0.9	Remove
29. Have release time or can accomplish innovations in residency training within their regular work load	3.8	1.2	Remove
30. The heavy workload reduces openness to educational change/innovation	3.9	1.1	Remove
**Staff culture**<br>Clinical staff members:			
31. Have a sense of personal responsibility for improving training	4.2	0.7	Stay
32. Cooperate to maintain and improve effectiveness of training	4.1	0.8	Stay
33. Are willing to innovate and/or experiment to improve training	4.1	0.7	Stay
34. Are receptive to changes in training methods	4.1	0.7	Stay
35. Have sufficient knowledge on change management to lead the implementation of this change ®	3.5	1.1	Remove
36. Have clearly defined roles and responsibilities with respect to residency training	3.6	0.8	Remove
37. Share responsibility for the success of this project	4.1	0.9	Stay
38. Work together as a team	4.2	0.8	Stay
39. Carry out the same opinion regarding this change towards trainees	3.8	1.0	Remove
40. Discuss this change with trainees in both formal and informal situations	3.9	0.8	Stay
**The formal leader of this innovation in residency training (e.g. the program director):**			
41. Accepts responsibility for the success of this project	4.1	0.9	Stay
42. Has the authority to carry out the implementation	4.4	0.8	Stay
43. Is considered an opinion leader	3.9	1.0	Remove

44. Cooperates well with the clinical staff members	4.4	0.8	Stay
**Involvement in this innovation in residency training:**			
45. There is good communication between formal educational leaders and us about the policy towards this change	4.0	0.8	Stay
46. Information provided on the change is clear	4.2	0.9	Stay
47. We are sufficiently informed about the progress of the change	3.9	0.9	Stay
48. We are consulted about the change sufficiently	3.9	0.9	Remove
49. We are informed about the reasons for the changes	4.0	0.9	Stay
Trainees are willing to innovate and/or experiment to improve training			New
**Project resources**<br>The following are available to successfully implement this innovation in residency training:			
50. Financial resources	4.1	0.9	Stay
51. Training	4.3	0.8	Stay
52. Facilities	4.1	0.9	Stay
53. Staffing	4.1	0.8	Stay
54. Staff incentives (e.g. financial reward, promotion)	3.6	0.8	Remove
55. Equipment and materials	4.0	0.8	Stay
56. Trainee awareness of this change	4.3	0.7	Stay
57. Incorporation of trainee needs	4.3	0.7	Stay
58. Evaluation protocol	4.1	0.8	Stay
**Clarity of mission and goals of this innovation in residency training.**			
59. We understand how this change fits as part of the desired competences of trainees	3.9	0.9	Stay
60. This curriculum change has clear goals and objectives	4.1	0.9	Stay
61. Our duties are clearly related to the goals of this change	3.9	0.9	Stay
62. Clinical staff has a clear plan for this change	3.9	0.9	Remove
**The implementation plan for this innovation in residency training:**			
63. Identifies specific roles and responsibilities	4.0	0.8	Stay
64. Clearly describes tasks and timelines	4.2	0.8	Stay
65. Includes appropriate training	4.0	1.0	Stay
66. Acknowledges clinical staff input and opinions	4.0	1.0	Stay
67. Includes a plan for improvement based on evaluations	4.1	0.9	Stay
